# Optimization of STP Innovation Management Mechanisms Driven by Advanced Evolutionary IoT Arithmetic

**DOI:** 10.1155/2021/1698089

**Published:** 2021-12-29

**Authors:** Tianxiang Wang, Qingqing Ma, Jinxi Li

**Affiliations:** ^1^Faculty of Management and Economics, Kunming University of Science and Technology, Kunming, Yunnan 650500, China; ^2^Anqing Normal University, Anqing, Anhui 246133, China; ^3^Party School of the CPC Xinyang Municipal Committee, Xinyang, Henan 464000, China

## Abstract

Since industrialization, manufacturing has been an important pillar of a country's economic development. Under the dual pressure of the new trend of global manufacturing development and the loss of competitive advantage of manufacturing industry, it is especially important to accelerate the enhancement of national high technology innovation capacity and the optimization of high technology policy innovation management mechanism driven by advanced evolutionary Internet of Things (IoT) arithmetic. The main of this paper thus introduces the effective method of optimization of high technology policy innovation management mechanism driven by advanced evolutionary IoT arithmetic. To study the optimization of high technology policy innovation management mechanism, a conceptual analysis of currently popular information technologies, such as big data technologies, artificial intelligence technologies, and Internet of Things technologies, and an overview of the application of these technologies in microgrids are given. In the paper, all factors are studied using the STP innovation management mechanism-based approach, and finally, all factors are classified into two categories of cause and effect factors by this approach, and the importance of all factors is ranked. Secondly, a wind power prediction algorithm based on data mining technology and an improved algorithm and a PV power prediction algorithm based on a deep neural network were established with the technical support of high-tech information technology such as big data and artificial intelligence. Finally, the majorization of high technology policy innovation management mechanism driven by advanced evolutionary IoT arithmetic is proposed.

## 1. Introduction

High technology innovation is the fundamental driving force of a country's development, and the high technology policy and innovation management mechanism of every country in the world today cannot hold a laissez-faire attitude towards high technology innovation in the process of economic development, especially in developed countries paying more attention to the development of high technology innovation [1], and their high technology policy and innovation management mechanism have been formulating a large number of high technology innovation policies to guarantee and promote the high technology innovation activities. Especially in the twenty-first century, mankind has entered the era of economic globalization, informatization, and networking, and the traditional economic development model has undergone significant changes. From the international perspective [2], an innovative economy will gradually become the mainstream form of economic development, innovation has become the focus of the development strategies of developed countries and regions, and innovative countries have become an important symbol of scientific and technological power. To gain long-term development advantages in the competition of economic globalization, scientific and technological innovation is imminent [3].

High technology is the source of a country's sustainable development, and high technology innovation is gaining ground in economic and social development, becoming a core element affecting national competitiveness. Improving the level of national high technology innovation has become the focus of attention of scholars and innovation management mechanisms of high technology policies in various countries [4]. For a long time, the leading countries in high technology have taken enterprises as the main body of high technology innovation, but the management mechanism of high technology policy innovation plays an important role in the process of promoting high technology progress. Because of the high risk and uncertainty of both market and technology in the process of development, and the monopoly of enterprises on scientific and technological achievements, all these situations will lead to market failure in the process of allocation of scientific and technological resources. Therefore, the role of the innovation management mechanism of high technology policy driven by advanced evolutionary IoT calculus is revealed. Innovation supports development and high technology lead the future [5].

Through the analysis of high technology policy innovation management mechanism high technology innovation policy orientation research, it can be seen that, in the rapid development of high technology today, to improve productivity and promote social progress at the same time, the need for continuous innovation, how to properly guide the innovation of high technology, and by whom to guide has become the current concern. The purpose of this paper is very clear for this topic, aiming to study the role played by high technology policy innovation management mechanism in the process of high technology innovation and how high technology policy innovation management mechanism promotes the formulation of high technology innovation policy. Optimize the index system of high technology innovation policy assessment. Clarifying the content of high technology innovation policy, exploring the main factors affecting the effect of high technology innovation policy, and constructing a reasonable and effective assessment index system are the prerequisites for correctly assessing the effect of high technology innovation policy. Based on the connotation and composition of high technology innovation policy, this paper selects indicators from three aspects, such as high technology innovation foundation and input, output and result, and regional economic benefit, and adopts factor analysis to screen the indicators, and finally determines the regional high technology innovation policy assessment indicator system, which is a useful supplement to the construction of high technology innovation policy effect assessment indicator system and provides a reference for the future high technology innovation policy effect assessment indicator system. It is a useful supplement to the construction of the STI policy effect assessment index system and provides a reference for the optimization of the future STI policy effect assessment index system.

## 2. Relevant Studies

After the introduction of “innovation” into economic theory for the first time in the literature, Western economists have explored the issue of innovation in greater depth, mainly from two perspectives: one is to introduce technological innovation as an exogenous variable into the production function to explain the role of technological innovation on economic growth; the other is to endogenize technological innovation [6]. It is believed that sustained economic growth does not rely on exogenous push, but endogenous technological progress. After the 1980s, a considerable number of studies on high technology innovation have been conducted, and their research is mainly based on the deepening and expansion of the endogenous economic growth theory, which contains organizational innovation, institutional innovation, and management innovation in addition to technological innovation. The literature studies innovation based on the management perspective and argues that energy creation is not enough to rely only on technological improvements, but also on other potential behaviors to improve the wealth creation of resources [7], such as management innovation. The literature includes institutional innovation as an endogenous variable in the innovation process and the empirical analysis of maritime transport productivity over the period 1600–1850 concludes that institutional improvements are more likely to cause productivity growth than technological changes [8]. Thus the literature argues that institutional innovation determines technological innovation rather than technological innovation determining institutional innovation. The literature investigates the role of income disparity in influencing the evolutionary path of innovative products and the returns to innovation, pointing out that the smaller the income disparity, the more conducive it is to stimulate innovative behavior, thereby promoting economic growth; conversely, it discourages innovative behavior and inhibits economic growth. The literature proposes the “quality of innovation,” which further elevates the previous criteria for judging innovation, such as novelty [9], unconventionality, and creativity, to the level of quality, i.e. standardization, systematization, and low variance. The literature argues that a firm's innovation capabilities include R&D, production, learning, marketing, resource development, organization, and strategic management. The literature explores the relationship between the price of innovative products and innovation behavior, stating that the price of innovative products is not an exogenous variable and that increasing the price of innovative products can increase the profitability of innovation when the income gap widens; on the other hand, a price increase can lead to a decrease in the rate of market demand, which in turn can reduce the return to innovation. The literature argues that innovation is a management process and that the scientific application of methods, rules, and institutions can be the efficient rate of completing innovative work [10].

The literature defines technological innovation based on the continuum of innovative behavior, design, manufacturing, and commercial activities involved in the process of producing new products, using new processes or new production equipment, and this activity process is in the order of process innovation, product innovation, and innovation diffusion [11]. The literature analyzes innovation from the perspective of the main position of enterprises and combines technological innovation with enterprises. It believes that innovation is a series of activities in which enterprises reallocate and combine production factors and conditions to obtain potential profit opportunities, rapidly capture market information, and establish highly efficient, effective, and low-cost production systems. In addition, the literature points out that technological innovation in a broad sense refers to the entire process from the conception of a new product or process to its development [12], technology diffusion, and realization of its market value. The literature suggests that technological innovation can be broadly defined as all actions from the conception of a new idea to the production of a new product, including not only the outcome of the innovation itself but also the introduction, diffusion, and application of the technology. The literature constructs an index system for evaluating scientific and technological capability from three perspectives: scientific and technological research and development capability, scientific and technological achievement transfer capability, and scientific and technological support capability, and conducts a longitudinal and cross-sectional comparative analysis of the scientific and technological innovation capability of City A [13].

Based on the endogenous economic growth theory, the literature introduces the endogenous knowledge stock into economic growth and measures the output of high technology activities by using high technology funding input, knowledge stock input, capital input and labor input, and human capital input as input indicators and explores the relationship and development trend between high technology input and high technology output performance in China. Based on the Cobb-Douglas production letter model with GDP as the dependent variable and labor input, capital input, and rate of scientific and technological progress as independent variables, the literature uses an econometric model to study the impact of scientific and technological progress on economic growth in country A during 1980–2004 and applies Solow's surplus to calculate the degree of contribution of scientific and technological progress to study the development of scientific and technological progress [14]. The literature argues that high technology innovation and economic development are complementary to each other, one is that high technology innovation drives rapid economic development, and the other is that economic development, in turn, provides strong support for high technology innovation. The literature adopts T-shaped correlation analysis to empirically study the contribution of economic investment in high technology and investment in scientific and technological talents in economic growth in country A during 2000–2007, and the results show that R&D investment has a very significant role in economic growth, while the role of scientific and technological talents in economic growth is not obvious. Economic growth is more dependent on the introduction of new technologies. In exploring the interaction between regional marine high technology innovation and blue economic growth, the literature points out to regional marine [15].

There is a high correlation of the growth of the regional blue-blue economy of ocean high technology innovation, and the relationship between them is not simply linear; it is necessary to establish a synergistic and efficient interactive operation mechanism to guarantee and support the positive interaction between the two and promote the coordinated development of regional marine high technology innovation and blue economy [16]. The literature discusses the mechanism of the role between high technology investment, high technology innovation, and regional economic development and proposes that there is a causal relationship between the three; i.e. high technology investment supports high technology innovation, and high technology innovation and regional economic development support each other, and high technology investment and regional economic growth are mutually reinforcing [17].

## 3. Optimization of STP Innovation Management Mechanisms Driven by Advanced Evolutionary IoT Arithmetic

### 3.1. Advanced Evolutionary IoT Arithmetic Example

The concept of the Internet of Things (IoT) was first proposed in 1991, and since then IoT has gradually entered people's vision. The Internet of Things uses Radio Frequency Identification (RFID), wireless communication, and other technologies to construct a physical Internet covering everything in the world based on the computer Internet. In the future, the continuous breakthrough of modern information technology will greatly promote the development of the Internet of Things, with the “Internet + Internet of Things,” “artificial intelligence + Internet of Things,” and other concepts of the perfect combination [18].

**Figure 1:**
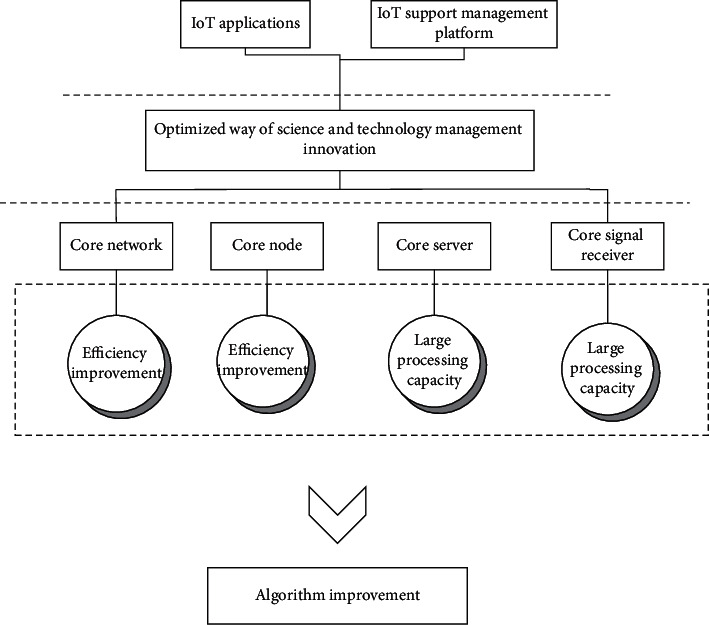
General framework of IoT.

The Internet of Things (IoT) is a kind of convergence application and enhancement after the development of modern information technology to a certain stage, based on RFID identification technology, to realize people and things, things and things can “communicate” with each other, forming an intelligent network that can sense the real world. In simple terms, it is mainly composed of two parts: the radio frequency tag and the decoder. The tag is attached to the surface of the item to be identified, and after entering the magnetic field, it receives the signal from the decoder and sends out the product information stored in the chip (passive tag or passive tag) by relying on the energy obtained from the induction current, or the tag actively sends out a signal of a certain frequency (active tag or active tag), and the decoder reads the information and decodes it and sends it to the central information system and finally performs relevant data processing and analysis. According to the three main aspects of IoT data processing, its logical functions are recognized as three levels, i.e., perception layer, network layer, and application layer. The perception layer mainly collects, identifies, and intelligently controls physical information in the real world; the network layer serves as a link between the perception layer and the application layer to guarantee the transmission, routing, and control between IoT information data; the application layer is the topmost layer in the three-layer structure, and its core functions include two aspects, one is to complete into the processing and management of various data, and secondly is the integration of these data with various industrial applications to achieve real-time control of the physical world. The general framework of the IoT is shown in Figure 1.

The current IoT technology mainly includes key technologies in the fields of perception and identification, intelligent control [19], embedded system, power supply, and energy storage, new material technology, wireless communication, chip design, and manufacturing, etc. The breadth of the involved fields and the complexity of the involved arts make it an extremely huge technical project. According to the presidential framework of IoT, the IoT technology system can be divided into three levels, such as perception layer technology, network layer technology, and application layer technology. Its formula principle lies in

(1) $$\begin{array}{c} \int \sqrt{d^{2}+x^{2}}=\frac{d\sqrt{b^{2}-4ac}}{2}. \end{array}$$

**Figure 2:**
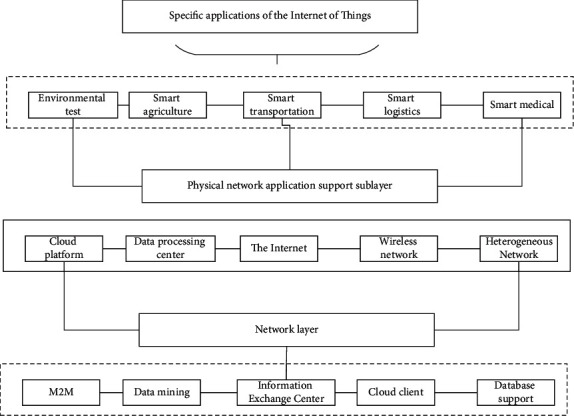
IoT technology architecture.

The first formula of the article summarizes the algorithm carried out in the article and summarizes the essence of the article. It is the basis of the algorithm mentioned in the article, and the following formulas are also mentioned. Among them, the sensing layer technology is the basis of the whole technical framework, the network layer technology is the technical guarantee of the whole information transmission, and the application layer technology is the terminal of the input and output control. The IoT technology system is shown in Figure 2.

The core technology of IoT is quite complex and fine, while the current overall R&D capability of China's IoT technology is weak, and when facing complex application requirements, it will be found that there is still a gap between the chip technology, hub components, and other high-precision technologies and the international leading level. Therefore, the development of IoT technology needs to be combined with China's specific national conditions and the current situation of industrial development to give priority to the development of IoT technology and provide a scientific basis for the country's macrodecision. At the same time, it is necessary to accelerate the development of the standardization of IoT technology [20] and steadily promote the promotion and application of IoT, to improve the overall level of IoT technology in China. With the rapid development of information technology, the scale of the IoT market will gradually expand, not only bringing huge economic benefits to society but also effectively safeguarding national security. IoT technology can be widely used in basic strategic areas such as security, electricity, food, oil, etc. It can also prevent the destruction of strategic resources such as strategic bases, public facilities, and forest land by hostile forces, terrorism, and natural disasters. The extensive use of IoT technology at the national strategic level provides a strong technical guarantee for securing homeland security. Its formula lies in

(2) $$\begin{array}{c} \int \sqrt{d^{2}+x^{2}}=\frac{1}{2}\frac{n!}{r!\left(n-r\right)!}+\sqrt{b^{2}-4ac}. \end{array}$$

**Figure 3:**
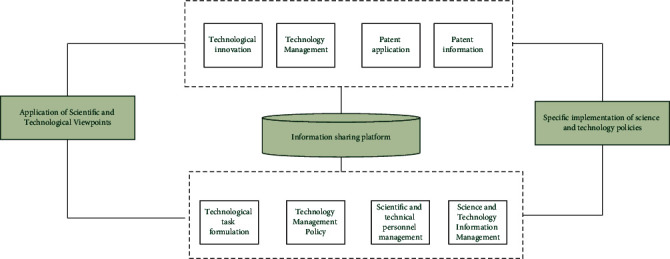
Diagram of industrial integration model.

The Internet of Things (IoT) applies a variety of modern information technologies to various industries, which itself is a complement and enhancement of the integration of two, which is also a component and application area of IoT technology. The concept of IoT is the integration of IT technology into automation control systems to form an intelligent network system that is efficient, safe, and energy-efficient. Using various advanced technologies such as perception, modern communication, artificial intelligence, and automation, the IoT reorganizes production factors and supply chains extensively and becomes a realistic carrier of informatization-driven industrialization. Therefore, the development of the Advanced Internet of Things Safety is a natural combination of industrialization and informatization, a powerful tool and substantial driving force to promote the integration of the two, and a realistic need to accelerate the integration of the two, whose realization model is shown in Figure 3.

### 3.2. Optimization of High Technology Policy Innovation Management Mechanism

High technology policy is the policy adopted by the government to promote the development of high technology and to use high technology to achieve national goals. Its policy texts are generally expressed in the form of circulars, plans, opinions, regulations, and laws and regulations. The theoretical basis of high technology policy includes three aspects, such as market failure theory, system failure theory, and scientific social contract theory. The objective of high technology policy is to promote economic and social development, the development of political civilization and national defense as well as the development of high technology, and the improvement of the country's comprehensive national power and international competitiveness. The high technology policy also contains the high technology talent policy in terms of the development of high technology talent. For the meaning of high technology policy, most scholars believe that high technology talent policy is a series of strategies, laws, regulations, decrees, ordinances, measures, and methods for managing and regulating the behavior of high technology talents. The high technology management system is the overall institutional arrangement of a country's high technology management, including the setting and management of high technology-related institutions, the process of high technology policy formulation and implementation, and many other elements, which plays an irreplaceable role in releasing national innovation vitality, improving the efficiency of the national research system, and maintaining the long-term stability of innovation.

There are several types of national S&T management systems: centralized, dualistic, and decentralized. Centralized systems usually involve the establishment of national ministries of high technology management at the national level to unify the country's high technology activities, and such unification usually facilitates the full protection and nurturing of research institutions and the conduct of long-term, high-risk research. However, scientific and technological development can easily run counter to actual needs or be distant from the mission of other government agencies, affecting the utilization of scientific and technological results. The decentralized system, in contrast to the centralized system, usually does not have a macro-high technology management ministry at the national level. This decentralized high technology management can ensure that each government ministry determines the meat of its *n* work according to its functional areas and management purposes and can respond to various problems promptly, and high technology development can be more *W* oriented to external needs and maintain high efficiency in the application of high technology results. The dual system is an intermediate type between centralized and decentralized, the national high technology system is generally managed through the national and regional administrative ministries of high technology education, high technology policy is mainly formed through a combination of a top-down and bottom-up approach, and the formation process is accompanied by the participation of many interest subjects, usually the product of the coordination of interests between the systems. With the development of high technology, international competition in high technology has become increasingly fierce, and the demand for innovation in countries has prompted countries to constantly adjust their internal decision-making institutions and organizational frameworks to meet their own internal innovation development needs and adapt to various new requirements. There are also many differences in S&T management systems among countries due to differences in political and economic systems. As one of the many functions of government, high technology management is as important as political, economic, cultural, and social management as a public management function of government. However, due to the special nature of high technology, there are many differences in the ways, systems, and procedures of management compared to other management functions of government.

**Figure 4:**
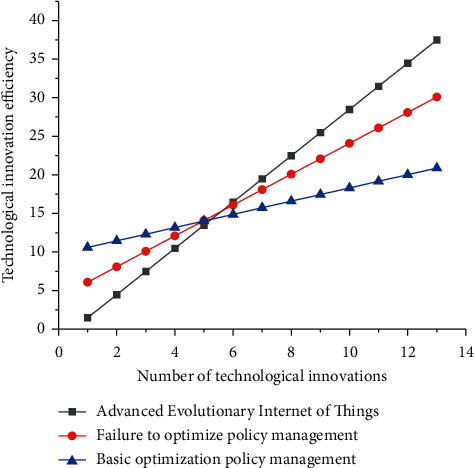
Optimized innovation efficiency display chart.

In summary, the optimal program of high technology management includes the following specific aspects: (1) The planning function. Planning is the prediction of future actions in light of the present situation. High technology planning refers to the actual situation of high technology development as the basis for the future implementation of high technology activities to develop a program. The planning function of government high technology management mainly refers to making plans for future high technology development work by clarifying the objectives of high technology development, reasonably allocating high technology resources, and indicating the direction for the development of high technology, to obtain the maximum promotion of high technology for social and economic development. The planning function of local government high technology management is that the local government, based on implementing and executing the national guidelines, policies, laws, and regulations on high technology development, makes plans, strategies, and policies for high technology development and high technology for economic and social development in a certain period in the future and formulates corresponding laws and regulations according to the actual situation of local high technology development. High technology plans are the fundamental guides to scientific and technological activities carried out by the main body of scientific and technological activities; to more effectively promote the development of local high technology, local governments at all levels have developed a plan for the development of high technology in line with the actual local situation and planning as the basis for scientific and technological activities to improve the level of local scientific and technological development, to better promote the development of the local wild economy. (2) Organizational functions. The organizational function of government high technology management is the premise and basis of its specific high technology management work, but also to achieve the goals of high technology management guarantee. The organization functions of local government high technology management means that the local government through the setting of the appropriate organizational structure, equipped with high technology management personnel, the provisions of high technology management departmental authority, and a clear relationship between the relevant ministries and personnel, achieve specific high technology management goals. Organizational functions are the preconditions for the realization of other high technology management functions, but also the basis for the orderly conduct of high technology management activities; local governments at all levels to better play the role of high technology management are actively improving its high technology management organizational functions, with the establishment of orderly high technology management operating mechanism to ensure the orderly implementation of government high technology management activities. (3) Coordination functions. The coordination function is an important part of the government's high technology management functions and is the guarantee of the smooth development of high technology activities. The coordination function of local government high technology management includes internal coordination and external coordination; internal coordination is the coordination between government high technology management related ministries, because the government's high technology management functions are scattered in a number of government ministries, in addition to the ministry in charge of high technology management; certain government ministries also have the function of high technology management; in order to ensure the orderly conduct of high technology management, it is necessary to coordinate the relationship between various ministries and agencies. External coordination is to coordinate the relationship between the government and other subjects of high technology activities; there are many subjects of high technology activities; in addition to the government ministries that undertake high technology management functions, universities, research institutions, enterprises, and intermediary organizations that provide services for government high technology management are all subjects of high technology activities. To ensure the smooth development of scientific and technological activities, we must coordinate the relationship between the subjects of scientific and technological activities and clarify the status of each subject in the management of high technology. The government is in a special position in many subjects of high technology activities and has the function of high technology management; therefore, the government must coordinate the relationship between the subjects of high technology activities to provide an orderly social environment for the development of local high technology. After undergoing optimization, its STI efficiency diagram is shown in Figure 4.

## 4. Experimental Results and Analysis

### 4.1. Experimental Results

**Figure 5:**
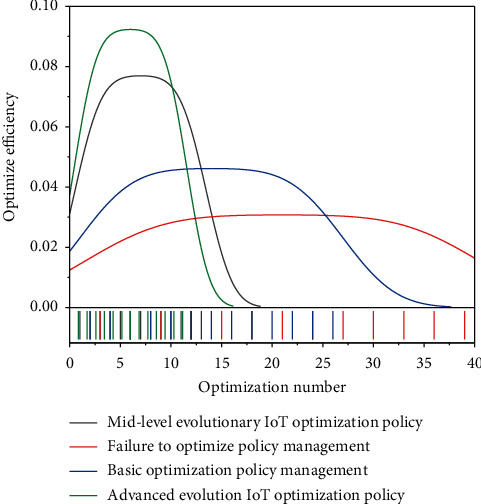
Comparison graph of experimental results.

The increase in the number of policy texts involving the departure and on-the-job entrepreneurship of scientific and technological personnel during the period of scientific and technological innovation indicates that the mechanism for the mobility and allocation of scientific and technological personnel in country A is gradually improving, but the proportion of policies on the management of scientific and technological personnel only accounts for 15% compared to other policy categories, indicating that the management of scientific and technological personnel is still weak in the system of scientific and technological personnel in the context of dual innovation, and the mechanism for the selection and use of scientific and technological personnel needs to be improved more. The problems that arise in the management mechanism of scientific and technological personnel are mainly the identity barriers of scientific and technological personnel leaving and starting a business on the job and the ambiguity of the policy of scientific and technological personnel innovation and entrepreneurship. Only by breaking the identity and geographical restrictions of scientific and technological personnel can China's human resources flow and be effectively allocated. Its efficiency diagram after the majorization of high technology management policies is shown in Figure 5.

**Figure 6:**
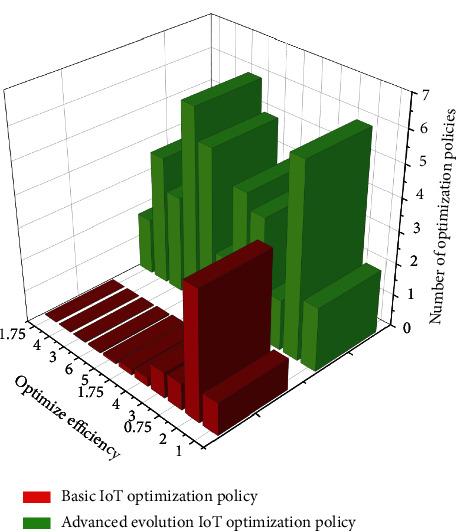
Trends in volume changes.

Subject to the traditional administrative management model, the government's high technology management work has the problem of overcontrolled thinking; the government's high technology management department in the management of high technology will see itself as the leading high technology management, trying to carry out a full range of high technology work, no omission of control. However, the government's high technology management function should be macroscopic guidance, the government's intervention is only to make up for the shortcomings of the market, in high technology activities to give full play to the role of the market to truly promote the development of high technology; the excessive intervention will only make the development of high technology deviate from the normal track, inhibiting the progress of high technology. Therefore, the local government in the management of high technology must establish the concept of service, do a good job in the management of high technology macrocontrol as the main task, and provide a good social atmosphere for the management of high technology, to provide services as the goal and pursuit. After its optimization, there is a significant improvement for high technology innovation, and its quantity changes as shown in Figure 6.

### 4.2. Experimental Analysis

To ensure the smooth implementation of high technology management, it is necessary to establish a sharing platform for high technology information, with the ministry in charge of high technology or a specialized agency responsible for summarizing and releasing high technology information on the information platform for sharing by high technology activity subjects. The first step in building a high technology information-sharing platform is to clarify the functional positioning of the high technology information-sharing platform, which should be a comprehensive, vertical, and horizontal exchange information system, and all information and resources related to high technology should be shared on the sending platform.

**Figure 7:**
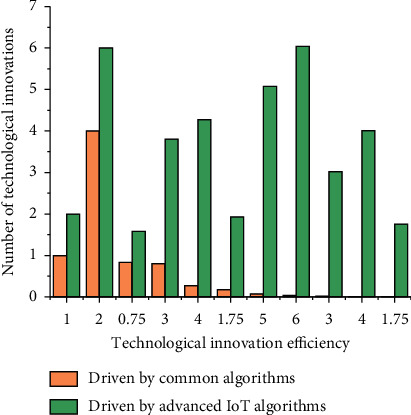
Trends in the efficiency of the mechanism system.

The government's high technology management department can understand the progress of local high technology work through the information-sharing platform, and other subjects of high technology activities can also understand the government's high technology trends through the information platform, to avoid the blindness of high technology activities and achieve the maximum benefit. Secondly, it is necessary to clarify the main body of the information-sharing platform. The information-sharing platform of high technology is not a way for government ministries to disclose information but should be an information platform jointly constructed by government ministries of high technology management, other relevant ministries, large high technology-based enterprises, universities, and institutes, and relevant enterprises and institutions. The high technology information released by the information platform should be all-rounded, with professional people ± responsible for classifying the high technology information to be released, forming a systematic information network, breaking the regionalization and compartmentalization of information, improving the efficiency of supervision, making up for the information asymmetry problems of ministries and related high technology industries, and eventually forming an information-sharing platform with the local high technology management ministry as the lead and other related high technology activity subjects as the cooperation. The trend in the efficiency of the system of mechanisms formed is shown in Figure 7.

**Figure 8:**
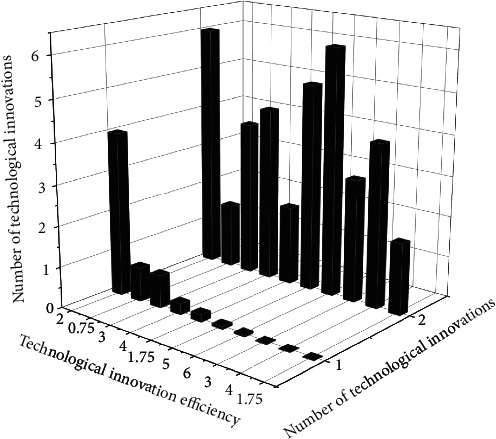
Efficiency trend performance graph.

Establishing a pluralistic decision-making advisory system, China's administrative decision-making has long relied mainly on official advisory bodies, and high technology decision-making advice is generally controlled by the Government's Ministry of High Technology Management, and the administrative tendency of decision-making advice is relatively serious. The purpose of establishing an expert consultation system for high technology decision-making is to improve the scientific nature of high technology decision-making, and only by ensuring the independence of the advisory body and establishing a diversified advisory system can the role of expert consultation be truly brought into play. The efficiency trends following the adoption of this method are shown in Figure 8.

## 5. Conclusion

High technology assessment is professional consultation and evaluation activity carried out by a specialized assessment agency by specific assessment requirements of the client, following certain assessment standards, procedures, and principles, and using scientific assessment methods to assess high technology policies, high technology plans, high technology projects, high technology results, and other relevant high technology activities that need to be assessed. Effective high technology assessment can improve the level of scientific research activities, ensure the quality of scientific and technological achievements, improve the efficiency of the use of scientific and technological resources, and provide a basis for the formulation of relevant high technology policies. A standardized and institutionalized assessment mechanism can greatly improve the quality of high technology assessment, and most developed countries have already built a relatively complete high technology assessment system and made the high technology assessment mechanism an essential part of high technology management and decision-making work.

High technology assessment can effectively guarantee the scientific nature of high technology management, and to give full play to the role of the assessment mechanism, the first thing is to improve the legal status of high technology assessment by way of legal regulation. One of the reasons why high technology assessment in developed countries can play a good role is that the legal status of high technology assessment is established in the form of laws, and the legal system of high technology assessment is promoted through the formulation of clear legal norms of assessment. Only then can the majorization of the management mechanism of high technology policy innovation driven by the advanced evolutionary IoT calculus reflect its advantages. The algorithm used in the article should pay attention to the efficiency of its operation in the subsequent development process, and the code used by the algorithm should use a more efficient algorithm language to improve the speed and efficiency of the algorithm.

## Data Availability

The data used to support the findings of this study are available from the corresponding author upon request.
